# Downregulation of the metalloproteinases ADAM10 or ADAM17 promotes osteoclast differentiation

**DOI:** 10.1186/s12964-024-01690-y

**Published:** 2024-06-11

**Authors:** Aaron Babendreyer, Julia Kieselhorst, Cindy Rinkens, Anastasia M. Lyashenko, Stefan Düsterhöft, Holger Jahr, Rogerio B. Craveiro, Michael Wolf, Andreas Ludwig

**Affiliations:** 1 Institute of Molecular Pharmacology, University Hospital RWTH Aachen, Pauwelsstr. 30, 52074 Aachen, Germany; 2 Institute of Anatomy and Cell Biology, University Hospital RWTH Aachen, Aachen, Germany; 3 Institute of Structural Mechanics and Lightweight Design, RWTH Aachen University, Aachen, Germany; 4 Department of Orthodontics, University Hospital RWTH Aachen, Aachen, Germany

**Keywords:** Osteoclast, Monocyte, Metalloproteinase, Shedding, Colony stimulating factor 1 receptor, Receptor activator of nuclear factor kappa-Β ligand, Knockout mice

## Abstract

Bone resorption is driven through osteoclast differentiation by macrophage colony-stimulating factor (M-CSF) and receptor activator of nuclear factor kappa-Β ligand (RANKL). We noted that a disintegrin and metalloproteinase (ADAM) 10 and ADAM17 are downregulated at the expression level during osteoclast differentiation of the murine monocytic cell line RAW264.7 in response to RANKL. Both proteinases are well known to shed a variety of single-pass transmembrane molecules from the cell surface. We further showed that inhibitors of ADAM10 or ADAM17 promote osteoclastic differentiation and furthermore enhance the surface expression of receptors for RANKL and M-CSF on RAW264.7 cells. Using murine bone marrow-derived monocytic cells (BMDMCs), we demonstrated that a genetic deficiency of ADAM17 or its required regulator iRhom2 leads to increased osteoclast development in response to M-CSF and RANKL stimulation. Moreover, ADAM17-deficient osteoclast precursor cells express increased levels of the receptors for RANKL and M-CSF. Thus, ADAM17 negatively regulates osteoclast differentiation, most likely through shedding of these receptors. To assess the time-dependent contribution of ADAM10, we blocked this proteinase by adding a specific inhibitor on day 0 of BMDMC stimulation with M-CSF or on day 7 of subsequent stimulation with RANKL. Only ADAM10 inhibition beginning on day 7 increased the size of developing osteoclasts indicating that ADAM10 suppresses osteoclast differentiation at a later stage. Finally, we could confirm our findings in human peripheral blood mononuclear cells (PBMCs). Thus, downregulation of either ADAM10 or ADAM17 during osteoclast differentiation may represent a novel regulatory mechanism to enhance their differentiation process. Enhanced bone resorption is a critical issue in osteoporosis and is driven through osteoclast differentiation by specific osteogenic mediators. The present study demonstrated that the metalloproteinases ADAM17 and ADAM10 critically suppress osteoclast development. This was observed for a murine cell line, for isolated murine bone marrow cells and for human blood cells by either preferential inhibition of the proteinases or by gene knockout. As a possible mechanism, we studied the surface expression of critical receptors for osteogenic mediators on developing osteoclasts. Our findings revealed that the suppressive effects of ADAM17 and ADAM10 on osteoclastogenesis can be explained in part by the proteolytic cleavage of surface receptors by ADAM10 and ADAM17, which reduces the sensitivity of these cells to osteogenic mediators. We also observed that osteoclast differentiation was associated with the downregulation of ADAM10 and ADAM17, which reduced their suppressive effects. We therefore propose that this downregulation serves as a feedback loop for enhancing osteoclast development.

**Supplementary Information:**

The online version contains supplementary material available at 10.1186/s12964-024-01690-y.

## Introduction

Infection or mechanical injury of the hard tissue can promote inflammation and bone loss. Bone loss is mainly mediated by osteoclastic bone resorption. Osteoclasts originate from the monocytic linage. The survival and proliferation of myeloid lineage cells are essentially driven by macrophage colony-stimulating factor (M-CSF) signaling through its receptor colony-stimulating factor 1 receptor (CSF1R, also known as M-CSFR, CD115 or c-FMS) [1, 2]. CSF1R is necessary but not sufficient for osteoclast differentiation. In addition, the receptor activator of NF-κB (RANK, also known as CD265) needs to be activated by the RANK ligand (RANKL) to complete osteoclast differentiation and activation [1, 2]. Both CSF1R and RANK are single transmembrane receptors that can be released from the cell membrane by limited proteolysis. This so-called shedding is mediated by metalloproteinase activity. The a disintegrin and metalloproteinase 17 (ADAM17) has been identified as one of the proteinases that shed both receptors [3, 4].

ADAM17 and the closely related protease ADAM10 are the major members of the ADAM family of metalloproteinases. Both proteinases are ubiquitously expressed in almost all cell types and cleave a variety of surface molecules from the cell surface. In doing so, they can control the release and activity of growth factors, cytokines and receptors to regulate cell proliferation, inflammatory responses and cell differentiation [5–7]. Some substrates are preferentially cleaved by either ADAM10 or ADAM17, but considerable overlap has been found in the substrate specificity of the two proteinases [8, 9]. Moreover, both proteinases clearly differ in their regulation mechanisms, resulting in the activation or inactivation of either proteinase. While ADAM10 function depends on TSPAN8 family members, ADAM17 requires the rhomboid pseudoproteinases iRhom1 and iRhom2 for maturation and surface expression. Whereas iRhom1 seems to be ubiquitously expressed, iRhom2 appears to play a role primarily in immune cells and in inflammatory processes [10–13]. This implies that substrate cleavage by either ADAM10 or ADAM17 may strongly depend on the cell type, its stimulation and differentiation status, and it cannot be predicted whether a substrate is cleaved by ADAM17, ADAM10 or both.

Since general knockout of ADAM10 or ADAM17 in mice results in severe developmental defects [14, 15], cell-specific conditional knockout mice for either proteinase have been studied. The critical role of the proteinases in inflammation and tumorigenesis has been demonstrated. Furthermore, cell-specific ADAM17-deficiency in chondrocytes was found to prevent ossification and long bone growth in mice by regulating terminal chondrocyte differentiation [16–18]. Another study revealed that conditional inactivation of ADAM17 in osteochondroprogenitor cells leads to osteoporosis [18]. ADAM10-deficiency in endothelial cells also negatively affects bone formation due to defects in vascular tree formation in endothelial cells [19]. In vitro results showed that ADAM10 siRNA-mediated knockdown led to reduced RANKL release by osteoblasts and subsequently impaired osteoclastogenesis in cocultured macrophages [20]. These findings suggest that ADAM10 and ADAM17 are both involved in bone remodeling. However, most of these effects are not direct, but instead involve the activity of cell types other than developing osteoclasts. It is therefore important to gain a clearer understanding of how ADAM10 and ADAM17 on osteoclast precursor cells specifically affect osteoclastogenesis. Proteolytic cleavage of the osteoclast receptors RANK and CSF1R by ADAM17 has been reported [3, 4]. Upregulation of ADAM17 in macrophages has been shown to disrupt osteoclastogenesis through shedding of CSF1R [21]. However, shedding of RANK and the relative contribution of ADAM10 to these shedding events have not been investigated. Furthermore, a possible correlation between the shedding of the two receptors, RANK and CSF1R, by both proteinases and osteoclast differentiation is not yet fully understood.

The aim of the present study was to examine the role of both ADAM17 and ADAM10 in shedding events during osteoclast differentiation in more detail. This was investigated by studying osteoclast differentiation in murine RAW264.7 cells, murine bone marrow derived monocytic cells (BMDMCs) and human peripheral blood mononuclear cells (PBMCs). First, we investigated the regulation of ADAM10 and ADAM17 expression during osteoclast differentiation. Then, both ADAMs were targeted by either inhibition or knockout. The effect on CSF1R and RANK shedding was correlated with the effect on osteoclast differentiation. We found that ADAM10 and ADAM17 are downregulated during osteoclast differentiation and that the loss of ADAM10 or ADAM17 activity promotes RANK and/or CSF1R surface expression and the induction of osteoclast differentiation.

## Methods

### Materials

The metalloproteinase inhibitor TAPI-1 was obtained from Selleck Chemicals LLC (Cat. No. S7434, Houston, USA). The preferential ADAM10 inhibitor GI254023X was synthesized and characterized as previously reported [22, 23]. M-CSF (Cat. No. 315-02) and RANKL (Cat. No. 315–11 C) were obtained from PeproTech (New Jersey, USA). CytoPainter Phalloidin-iFluor 594 Reagent was obtained from Abcam (Cat. No. ab176757, Cambridge, GB). 4’6, Diamidino-2-phenylindol dihydrochloride (DAPI) was obtained from Sigma‒Aldrich (Cat. No. D9542, Steinheim, Germany). Monoclonal antibodies against murine CD115/c-Fms/M-CSFR/CSF1R (Clone AFS98, Cat. No. 14-1152-81) and murine CD256/RANK (Clone R12-31, Cat. No. 14-6612-82) were obtained from Thermo Fisher Scientific (Waltham, USA). The rat IgG2A isotype control (Cat. No. MAB006) was obtained from R&D Systems (Minneapolis, USA). An allophycocyanin (APC)-conjugated secondary antibody was obtained from Jackson (Cat. No. 112-136-071, Suffolk, GB).

### Mice

The use mice or isolation of bone marrow cells is in compliance with the rules of the EU Directive 2010/63/EU and the local committee LANUV NRW, has been notified (notification 5016A4). Wild -type C57Bl/6J mice were from in house breeding. For knockout of ADAM17 in hematopoietic cells, C57Bl/6J mice (Jackson, Bar Harbor, US) transgenic for Cre recombinase under the control of the promoter for guanine nucleotide exchange factor 1 (Vav) were crossed with C57Bl/6J mice carrying floxed ADAM17. Genotyped offspring carrying one allele of Cre recombinase and two alleles of floxed ADAM17 were used for ADAM17 knockout [24]. The absence of ADAM17 protein was confirmed in a separate study [25]. Mice that carried Cre recombinase but no floxed proteinase alleles were used as littermate controls expressing ADAM17. iRhom2Del mice were generated by electroporation of C57BL/6 N zygotes with Cas9 protein (IDT) synthetic guide RNA and a single-stranded repair template to introduce a deletion of 13 nucleotides (Δ13: TGGGCCCCACTGG) resulting in an early translational stop. Zygotes were implanted into foster mothers; newborn founder animals were routinely genotyped and absence of iRhom2 protein in Bone marrow derived macrophages was confirmed by Western Blotting [12].

### RAW264.7 cell culture and differentiation into osteoclasts

The murine macrophage line RAW264.7 was cultured in DMEM supplemented with high glucose (4.5 g/l glucose) without antibiotics in 24-well cell culture plates. Different lots of fetal bovine serum (FBS, Gibco, Thermo Fisher Scientific) were tested for optimal results in osteoclast differentiation and then used at a final concentration of 10%. The cells were subcultivated at 70% confluence by removal from the dish with a soft cell scraper until a final total passage number of 11. For osteoclastic differentiation, 7,000 cells per well of a 24-well plate were seeded and after 24 h stimulated with 100 ng/ml murine RANKL (day 0). Optimal RANKL concentration of 100 ng/ml was evaluated by testing a concentration series ranging from 10 to 200 ng/ml RANKL as suggested by Marino et al. [26]. After an additional 3 days, the medium and RANKL were renewed. From day 4 on, the cells first formed protrusions and fused into large multinucleated cells, and the greatest number of osteoclasts was reached on day 5.

### Monocytic cell isolation from murine bone marrow and human peripheral blood and osteoclast differentiation

Murine BMDMCs were isolated from the femur and tibia of the hind limbs of 8- to 12-week-old mice as previously described [27]. After erythrocyte lysis, 25,000 cells were seeded per well of a 24-well plate, and after 3 h, the nonadherent cells were removed. Subsequently, adherent cells were stimulated with 30 ng/ml murine M-CSF for 7 days, and the medium was changed on days 3 and 5, followed by the addition of 100 ng/ml murine RANKL. Optimal concentrations for RANKL and M-CSF were evaluated by testing a concentration series ranging from 10 to 200 ng/ml for RANKL and 20–50 ng/ml for M-CSF as previously recommended [26]. The medium was changed on day 9 and day 11, and the medium volume was increased from 500 µl to 1000 µl. After day 9–12, when multinucleated giant cells were visible, the cells were stained, or RNA was extracted for quantitative PCR (qPCR) analysis.

Experiments with human mononucleated of healthy from healthy donors were in compliance with the Helsinki declaration and approved by the local ethics committee of the University Hospital RWTH Aachen (vote EK172/10). Cells were isolated from mononucleated cells were isolated from citrated peripheral blood of healthy donors by centrifugation on a Pancoll gradient (Cat. No. P04-60500, PAN Biotech, Aidenbach, Germany) as previously described [27]. PBMCs were seeded in α-MEM (Minimum Essential Medium Eagle Alpha Modification, Cat. No. M4526, Sigma-Aldrich) at 2 × 10^6^ per well of a 24-well plate. After 4 h, the cells were washed once, and adherent monocytic cells were stimulated with 30 ng/ml human M-CSF. The medium was changed on days 3, 6, 9, 12 and 15. From day 6 on, the cells were stimulated with M-CSF with or without additional human RANKL (75 ng/ml), and from day 12, the medium volume was increased from 500 µl to 1000 µl. On day 17, differentiation was assessed by staining and qPCR.

### Osteoclast staining

The Leukocyte Acid Phosphatase Kit (Cat. No. 387 A, Sigma-Aldrich) was used to detect tartrate-resistant acids in osteoclasts. After two washes with PBS, the cells were fixed using Roti-Histofix 4% (Cat. No. P087.3, Carl Roth, Karlsruhe, Germany) for 10 min at RT and then washed again with PBS. To permeabilize the cells, a 5-minute incubation with 0.1% Triton X-100 in PBS was followed by a wash step with PBS. The plates were then incubated for 1 h at 37 °C in 1 M Tris-HCl buffer (pH 7.4). For staining, GBC solution was first prepared from Fast Garnet GBC and sodium nitrite solution (1:1) by careful inversion. After 2 min, the GBC solution was used to prepare the TRAP staining solution. For a full 24-well plate, this consisted of 250 µl of GBC solution, 11.25 ml of distilled water, 125 µl of naphthol AS-Bi naphthol solution, 500 µl of acetate solution and 250 µl of tartrate solution. The cells were then incubated for 1 h in TRAP staining solution at 37 °C. The stained cells were washed once with tap water and once with distilled water and stored dry.

For staining of actin filaments fixed cells were incubated with CytoPainter Phalloidin-iFluor 594 Reagent (Abcam) at room temperature for 40 min according to the manufacturer’s protocol and then washed with distilled water. Cell nuclei were visualized using the cell-permeable fluorescent dye DAPI (Sigma-Aldrich). Therefore, the cells were additionally incubated for 5 min with 200 µl of 5 µM DAPI in PBS at room temperature and then washed with distilled water. The TRAP staining of *RAW264.7* cells was analyzed using the ImageJ (version 1.53t, NIH, USA) plugin Weka Trainable Segmentation (version 3.2.34) [28], which is based on supervised machine learning. For this purpose, a classifier was trained to recognize the TRAP-positive regions, which were subsequently quantified using ImageJ and presented as a percentage of the total area (Fig. 3D). Due to the significant variation in TRAP staining of BMDMCs and PBMCs, automated quantification, as with the RAW264.7 cells, was not feasible. In these cases, the contours of TRAP-positive cells with at least three nuclei were manually drawn and subsequently quantified using ImageJ and presented as number of osteoclasts, total area of osteoclasts, and average size of osteoclasts (total area divided by the number).

### RNA extraction, cDNA synthesis and qPCR analyses

RNA was extracted using the RNeasy Mini Kit (Cat. No. 74,106, Qiagen, Hilden, Germany). The cells were washed with PBS and then lysed with 350 µl of RLT buffer. For experiments with RAW264.7 cells, the cells from one well of a 24-well plate were used to generate lysates. For lysates from primary cells, the cells from three wells of a 24-well plate were pooled. QIA shredder columns were used to homogenize the lysates. RNA isolation from cell lysates was performed according to the manufacturer’s protocol. The isolated mRNA was quantified photometrically (NanoDrop, Peqlab, Erlangen, Germany). RNA (350 ng) was reverse transcribed into cDNA using PrimeScript™ RT Master Mix (Cat. No. RR036B, Takara Bio Europe, St-Germain-en-Laye, France) and diluted 1:10 in nuclease-free water.

PCRs were performed using iTaq™ Universal SYBR® Green Supermix (Cat. No. 1,725,124, Bio Rad, Hercules, CA, USA) according to the manufacturer’s protocols. The primers used and their corresponding annealing temperatures can be found in the supplemental material (Suppl. Table 1). PCRs were run on a CFX Connect Real-Time PCR Detection System (Bio-Rad) with 45 cycles of denaturation for 10 s at 95 °C, followed by annealing for 10 s and amplification for 15 s at 72 °C. The efficiency of each qPCR run was calculated as the mean efficiency of all samples that fulfilled certain requirements with linear regression using LinReg software (v2018.0, Dr. J.M. Ruitjer, Academical Medical Centre Amsterdam, The Netherlands). The following exclusion criteria were used: (1) no amplification, (2) no plateau, (3) noisy sample, and (4) PCR efficiency outside 5% of the group median. Relative quantification was performed using CFX Maestro software (1.1, Bio-Rad). A reference gene analysis with 10 different murine reference genes was performed using CFX Maestro 1.1 software to determine the two most stable reference genes for the experimental setup. As a result, Gapdh and Eef2 were chosen for the RAW264.7 cells, and Gapdh and Rps29 were chosen for the BMDMCs.

### Flow cytometry

Flow cytometry was used to analyze the surface expression of CSF1R and RANK on RAW264.7 cells and BMDMCs. The cells were detached using a soft cell scraper, and 0.2 × 10^6^ cells were washed once and fixed with 2% PFA in PBS for 5 min on ice. After centrifugation, the cell pellet was incubated with the corresponding primary antibody or isotype control for 45 min on ice. After two washes, the cells were incubated in secondary antibody solution for 45 min. After two washes, the fluorescence signals were recorded by flow cytometry (LSR Fortessa, BD Bioscience, Heidelberg, Germany). The data were analyzed with FlowJo 10.2 software (Tree Star, Inc., Ashland, USA). To determine the surface expression, the geometric mean of the fluorescence intensities was determined after subtracting the nonspecific signal of the isotype control.

### Statistics

Quantitative data are given as the mean plus standard deviation (SD) calculated from a minimum of three independent experiments. Statistics on homoscedastic data were performed using generalized mixed model analysis (PROC GLIMMIX, SAS 9.4, SAS Institute Inc., Cary, North Carolina, USA) and assumed to be from either a normal, beta, negative binomial or lognormal distribution with the day of the experiment conducted as a random effect to assess differences in the size of treatment effects across the results. Analytical residual plots and the Shapiro‒Wilk test were used for determining homoscedasticity and a normal distribution. The degrees of freedom within the model were estimated by the Kenward–Roger approximation. In the case of heteroscedasticity, a nonparametric Kruskal–Wallis test was used. The analysis was always performed with non-normalized raw data. All p-values were adjusted for multiple comparisons by the false discovery rate (FDR). All p-values < 0.05 were considered significant.

## Results

### ADAM10 and ADAM17 expression is downregulated during osteoclastic differentiation of RAW264.7 cells

**Fig. 1:**
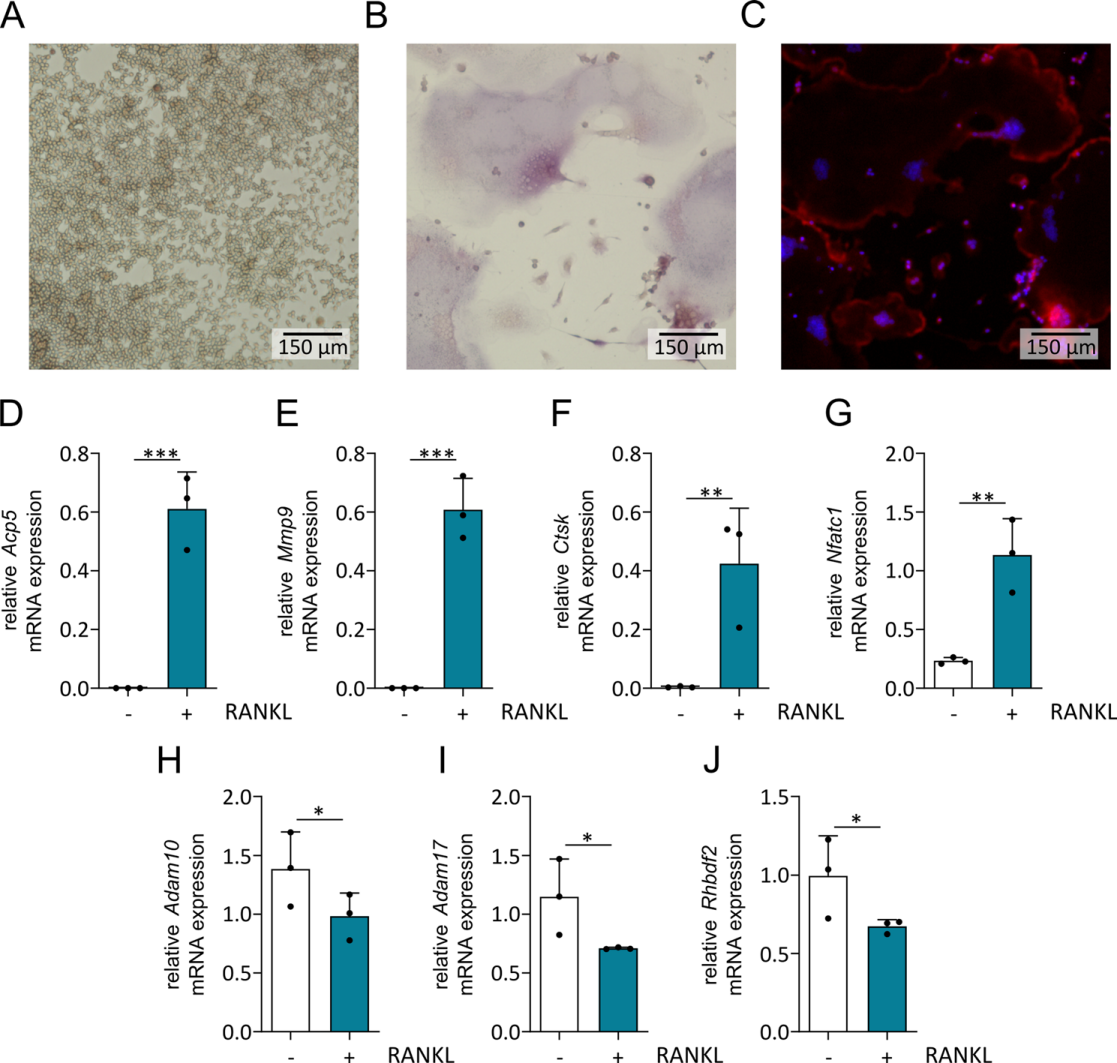
Osteoclastic differentiation and regulation of mRNA expression in RAW 264.7 cells in response to RANKL. RAW 264.7 cells were stimulated with RANKL for 5 days or left unstimulated. (**A**–**C**) After fixation and permeabilization, the cells were stained for TRAP and with DAPI and phalloidin. Representative images of TRAP staining of (**A**) unstimulated cells, (**B**) stimulated cells and (**C**) the corresponding fluorescence images of stimulated cells are shown. (**D**–**I**) After 4 d of stimulation of RAW 264.7 cells with and without RANKL, the mRNA expression of the osteoclast-associated marker genes Acp5/TRAP (**D**), Mmp9 (**E**), Ctsk (**F**) and Nfatc1 (**G**) as well as Adam10 (**H**), Adam17 (**I**) and Rhbdf2 (**J**) was determined by qPCR. The expression of these genes was related to that of the reference genes Eef2 and Gapdh. Quantitative data points are shown for each independent experiment and are summarized as the mean + SD. Significant differences are indicated by asterisks (* *p* < 0.05, ** *p* < 0.01 and *** *p* < 0.001)

We first studied the osteoclastic differentiation of the murine monocytic cell line RAW264.7. For the induction of osteoclast differentiation, cells were stimulated with 100 ng/ml RANKL. Beginning on day 4, the cells formed protrusions and then fused into large multinucleated cells. On day 5, the maximal number of osteoclasts was obtained. These cells were identified as osteoclasts by staining for tartrate-resistant acid phosphatase (TRAP) (Fig. 1A - B). Additionally, immunofluorescence staining with phalloidin and DAPI demonstrated the characteristic formation of actin rings and the presence of multiple nuclei (Fig. 1C). Quantitative PCR revealed the upregulation of osteoclast marker genes, such as *Acp5* (coding for the tartrate-resistant acid phosphatase), *Mmp9* (matrix metalloproteinase 9), *Ctsk* (cathepsin K) and *Nfatc1* (nuclear factor of activated T cells 1) (Fig. 1D - G). Moreover, we observed that the mRNA expression of *Adam10, Adam17* and the ADAM17 regulator *Rhbdf2* (iRhom2) in RANKL-stimulated cells was lower than in unstimulated cells (Fig. 1H - J). These results prompted the question of whether the downregulation of the proteinases is functionally linked to the induction of osteoclast differentiation due to altered shedding activity of ADAM10 or ADAM17.

### ADAM17 or ADAM10 inhibition promotes the osteoclastic differentiation of RAW264.7 cells

**Fig. 2:**
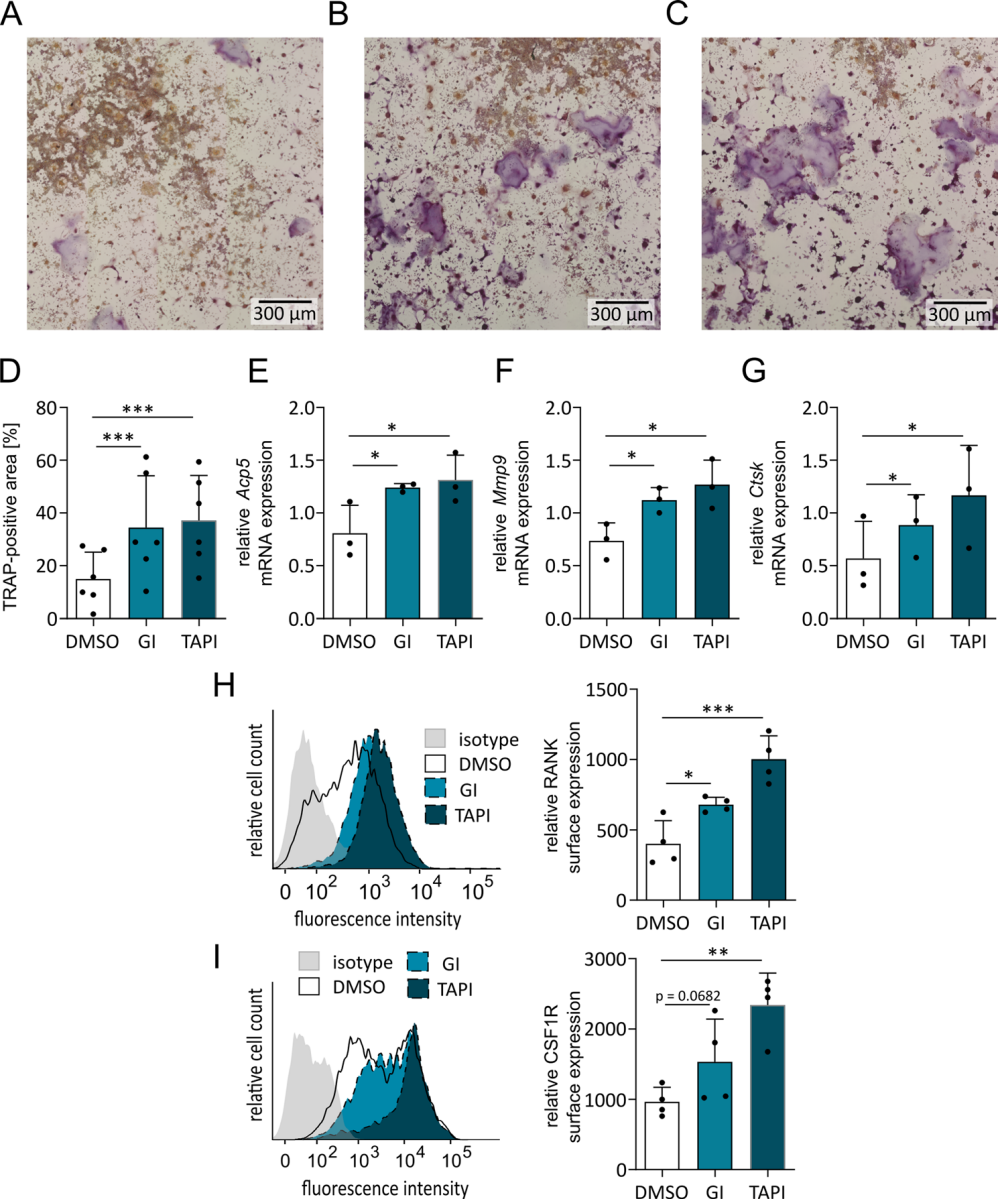
Effect of ADAM inhibitors on the osteoclastic differentiation of RAW 264.7 cells. (**A**–**D**) RAW 264.7 cells were stimulated with RANKL in the presence of the vehicle control DMSO (**A**), 10 µM GI (**B**) or 20 µM TAPI (**C**). TRAP staining was performed after 5 d. Representative images are shown. Osteoclast differentiation was quantified as the area of TRAP-positive cells (**D**). (**E**–**G**) After 4 d of stimulation with RANKL, the mRNA expression of the osteoclast-associated marker genes Acp5/TRAP (**E**), Mmp9 (**F**) and Ctsk (**G**) was determined by qPCR. (**H**, **I**) After 2 days, the surface levels of RANK (**H**) and CSF1R (**I**) were assessed by flow cytometry. Representative histograms of fluorescence intensities are shown. To quantify surface expression of RANK and CSF1R, geometric mean values of fluorescence intensities were determined and nonspecific binding of an isotype control antibody was subtracted. Quantitative data are presented as the mean + SD of independent experiments, and significant differences are indicated by asterisks (* *p* < 0.05, ** *p* < 0.01 and *** *p* < 0.001)

Next, we questioned whether reduction of ADAM10 and ADAM17 expression is relevant for osteoclastogenesis, and inhibition of the remaining proteinases could further enhance osteoclast development. To test this hypothesis, the effects of two inhibitors on RAW264.7 cell differentiation into osteoclasts were studied. GI254023X (GI) was used as a preferential ADAM10 inhibitor, with 100-fold lower potency for ADAM17 [29]. TAPI-1 (TAPI) was used as an established preferential ADAM17 inhibitor with approximately 10-fold lower potency for ADAM10 [30]. After 5 d of RANKL stimulation, TRAP staining revealed that compared with vehicle control, GI or TAPI treatment significantly increased osteoclast differentiation (Fig. 2A - D). Additionally, on day 4 of inhibitor treatment significant upregulation of osteoclast marker gene expression was observed compared to the vehicle control group (Fig. 2E - G). These results indicate that not only ADAM17 but also ADAM10 activity can limit osteoclast differentiation.

### ADAM17 or ADAM10 inhibition increases the surface levels of RANK and CSF1R

To address the functional mechanism by which ADAM10 and ADAM17 influence osteoclast differentiation we determined the surface expression levels of RANK, which is the receptor for RANKL. RANK is a known substrate of ADAM17 [3]. In the absence of ADAM17, the receptors accumulate on the cell surface and thus increase the sensitivity of cells to RANKL. In fact, RANK surface expression was higher in cells treated with GI or TAPI than in cells of the vehicle control group (Fig. 2H). In parallel, we also studied the surface expression of the M-CSF receptor (CSF1R), since RAW264.7 cells are well known to produce M-CSF in response to RANKL stimulation [31], leading to autocrine stimulation via CSF1R. This receptor is another ADAM17 substrate and therefore could also be linked to the inhibition of osteoclast differentiation by ADAM17 [32]. The presence of the inhibitors resulted in an enrichment of the cell population with high CSF1R surface expression (Fig. 2I). This effect reached significance for TAPI-1, and for GI stimulation the p-value was 0.0682. Overall, the effect of the ADAM17 inhibitor TAPI-1 seemed stronger than the effect of the ADAM10 inhibitor GI for both receptors. Nevertheless, the surface expression levels of CSF1R and RANK are controlled not only by ADAM17, as previously reported but also by ADAM10.

### ADAM10 and ADAM17 are downregulated during osteoclastic differentiation of primary murine macrophages

**Fig. 3:**
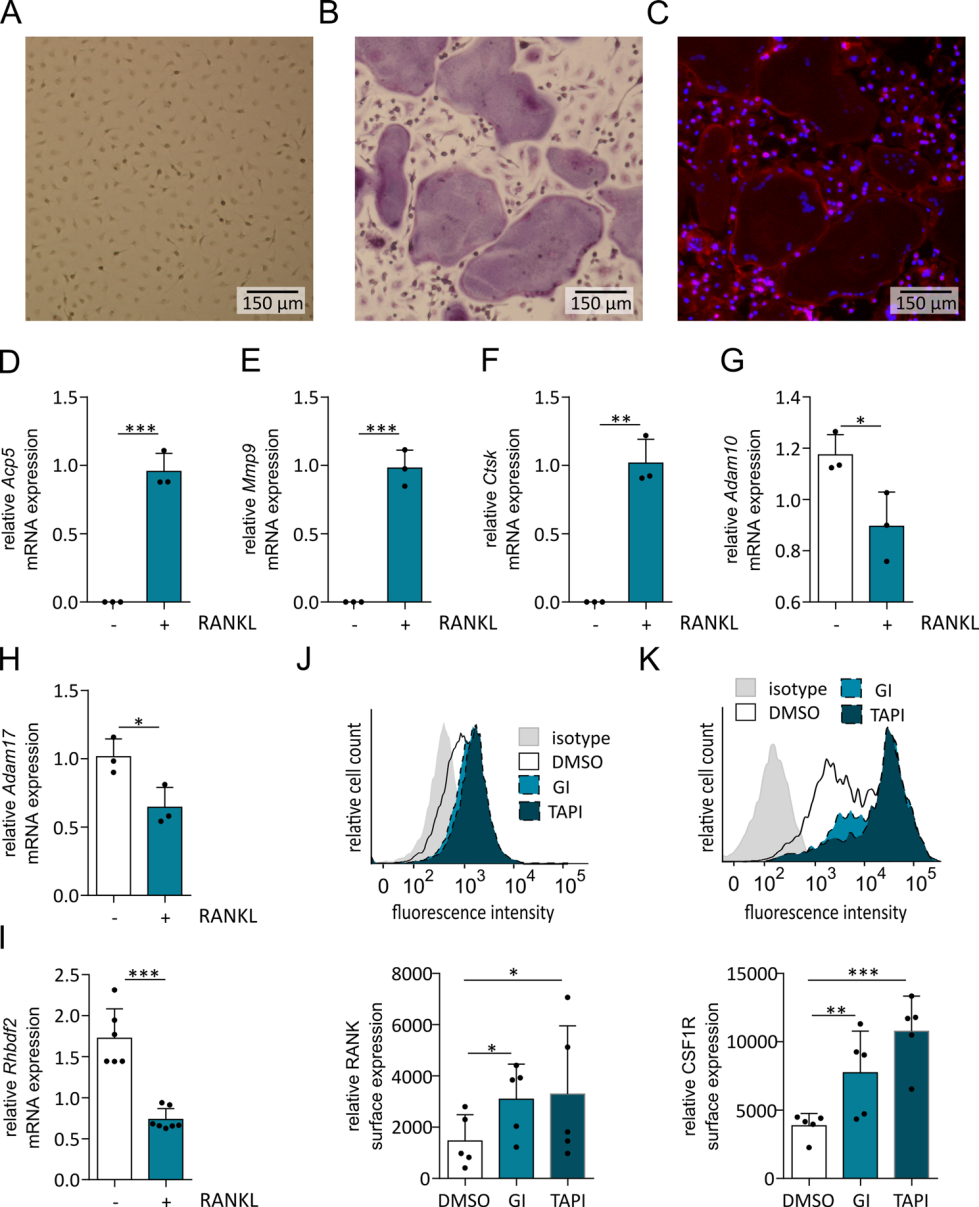
Osteoclastic differentiation of BMDMCs and regulation of RANK and CSF1R surface expression levels. (**A**–**C**) Isolated BMDMC from mice were differentiated into osteoclasts by stimulation for 7 d with M-CSF and subsequently for 5 d with additional RANKL. Cells that were only treated with M-CSF served as a control. After 12 d, the cells were stained for TRAP together with DAPI and phalloidin. Representative images of TRAP stained murine BMDMCs treated with M-CSF (**A**) or with both M-CSF and RANKL (**B**) and the corresponding fluorescence image for B (**C**) are shown. (**D**–**I**) The mRNA expression of the osteoclast-associated marker genes Acp5/TRAP (**D**), Mmp9 (**E**) and Ctsk (**F**) as well as that of Adam10 (**G**), Adam17 (**H**) and Rhbdf2 (**I**) was determined by qPCR. The expression of these genes was related to that of the reference genes Gapdh and Rps29. (**J**, **K**) BMDMCs were stimulated with M-CSF in the presence or absence of GI or TAPI. After 7 d, the surface levels of RANK (**J**) and CSF1R (**K**) were assessed by flow cytometry. The quantitative data are presented as the means + SD of independent experiments, and significant differences are indicated by asterisks (* *p* < 0.05, ** *p* < 0.01 and *** *p* < 0.001)

To confirm our results with primary cells we used murine monocytic cells obtained from the bone marrow of wild-type mice. Wild-type BMDMCs were stimulated with M-CSF for 7 days and subsequently differentiated into osteoclasts by stimulation with RANKL. Cells that were stimulated with M-CSF but not with RANKL served as control. After 12 days, the cells were fixed, permeabilized and stained for TRAP. The RANKL-stimulated cells showed a strong increase in size and TRAP staining (Fig. 3A, B). Nuclear staining with DAPI also revealed the presence of at least 3 nuclei and the formation of typical actin rings was confirmed using phalloidin (Fig. 3C). The mRNA expression of typical osteoclast markers was upregulated (Fig. 3D - F), whereas the mRNA expression of *Adam1, Adam17* and *Rhbdf2* was downregulated upon treatment with RANKL (Fig. 3G - I). Next, we studied the effect of GI and TAPI-1 on the expression levels of RANK and CSF1R. After 7 days of treatment together with M-CSF, both inhibitors enhanced the surface expression of RANK and CSF1R (Fig. 3J, K). These findings are consistent with the results obtained for RAW264.7 cells and further indicate a negative association of ADAM10 and ADAM17 with osteoclastogenesis.

### ADAM17 or iRhom2 deficiency increases osteoclastic differentiation from primary murine monocytic cells

**Fig. 4:**
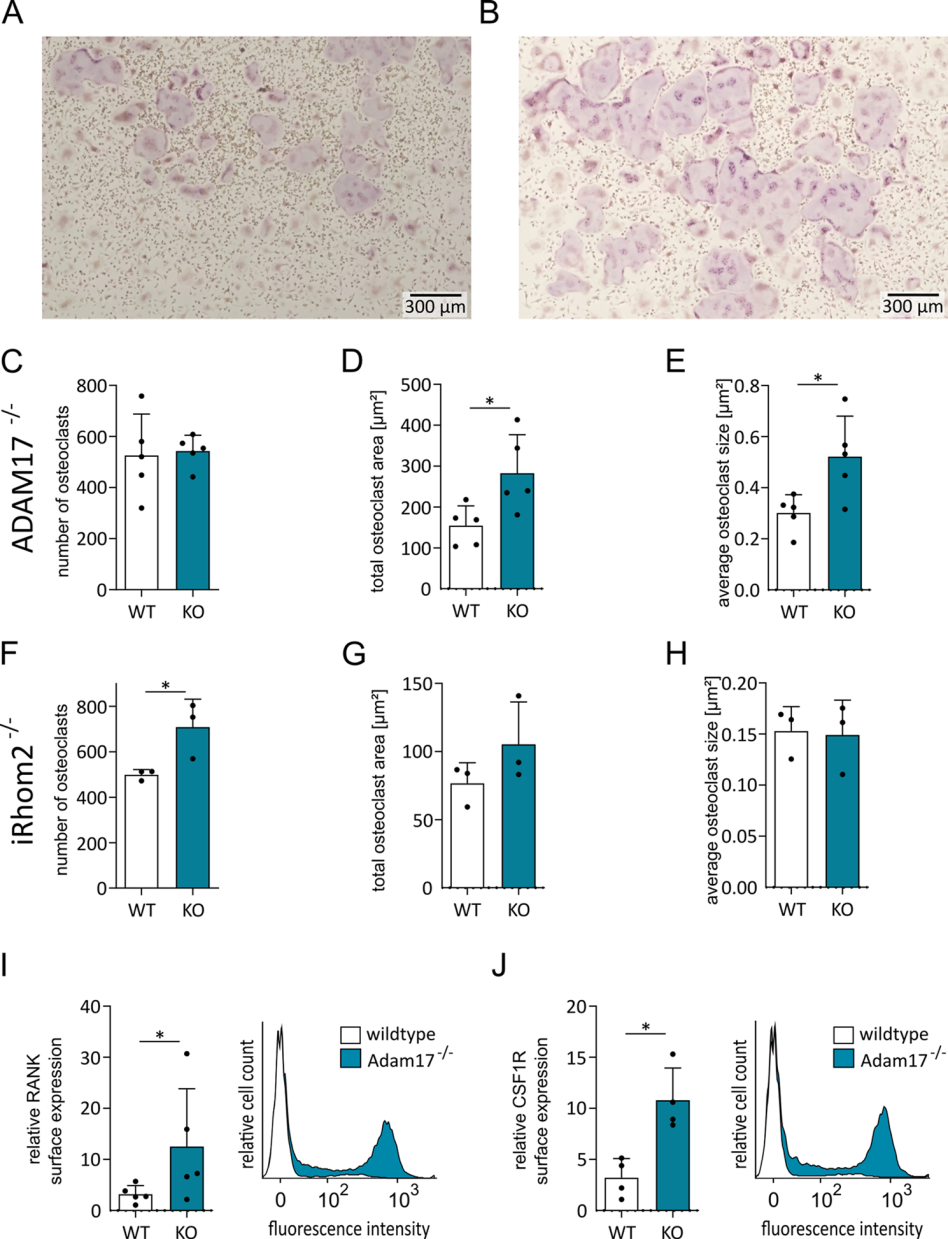
Effect of ADAM17 and iRhom2 knockout on osteoclastic differentiation and surface levels of RANK and CSF1R on BMDMCs. (**A**–**H**) ADAM17- (**A**–**E**) or iRhom2-deficient (**F**–**H**) BMDMCs (KO) and BMDMCs from corresponding wild-type (WT) mice were stimulated with M-CSF on day 0 and subsequently with additional RANKL on day 7. After day 11, the cells were fixed and stained for TRAP. Representative images of the osteoclastic differentiation of WT (**A**) and ADAM17-deficient BMDMCs (**B**) are shown. For quantitative analysis, the number and total area of TRAP-positive cells were determined per image and are displayed as data points for each independent experiment (**C**, **D**, **F**, **G**), and the average osteoclast size was calculated from the total osteoclast area divided by the number of osteoclasts (**E**, **H**). The surface levels of RANK and CSF1R on WT or ADAM17-deficient BMDMCs were assessed at day 7 of differentiation by flow cytometry (**I**, **J**). The quantitative data are presented as the means + SD of independent experiments, and significant differences are indicated by asterisks (* *p* < 0.05, ** *p* < 0.01 and *** *p* < 0.001)

Next, we used BMDMCs from mice with Vav-Cre-driven cell-specific ADAM17 deficiency in hematopoietic cells to study the role of ADAM17 in osteoclast differentiation in more detail. In general, these mice exhibited normal breeding behavior and showed no obvious differences in bone size. However, these mice had a different genetic background than the mice used for BMDMC preparation in previous experiments. Since the genetic background can influence osteoclast differentiation [33, 34] we repeated the above experiment with BMDM from wild-type animals of the Vav-Cre ADAM17 breeding. Osteoclast differentiation of BMDMCs in response to M-CSF and RANKL stimulation was evaluated by TRAP staining. We observed a similar induction of osteoclastogenesis and a similar trend for the downregulation of *Adam10* and *Adam17* during osteoclastogenesis (suppl. Figure 1). BMDMC were then prepared from Vav-Cre ADAM17 deficient animals and compared to BMDMC from wild-type (WT) animals. Although the number of developing osteoclasts did not change in the absence of ADAM17, the size of ADAM17-deficient osteoclasts was considerably larger than that of osteoclasts from wild-type (WT) animals (Fig. 4A - E).

Along with the downregulation of *Adam17*, we observed a downregulation of the critical ADAM17 regulator iRhom2/*Rhbdf2* after RANKL treatment of RAW 264.7 cells and BMDMCs from wild-type mice (Figs. 1J and 3I). Therefore, we also investigated the ability of BMDMCs prepared from mice with a complete iRhom2 knockout to induce osteoclast differentiation via M-CSF and RANKL. Consistent with the results for ADAM17-deficient BMDMCs, iRhom2 deficiency also positively affected osteoclast formation. However, iRhom2 deficiency led to an increase in the number of osteoclasts rather than an increase in their size, as observed for ADAM17-deficient BMDMCs. This difference may be due to differences in the mouse strains leading to differences in the kinetics of osteoclast precursor proliferation and subsequent fusion. This was already indicated by the smaller cell size of the wild-type controls in the iRhom2 knockout experiment than in the ADAM17 knockout experiment (Suppl. Figure 2A, B, Fig. 4F – H).

### ADAM17 deficiency increases RANK and CSF1R surface expression on osteoclast precursors

To study the possible influence of RANK and CSF1R shedding by ADAM17, the surface levels of RANK and CSF1R were assessed by flow cytometry after 7 days of stimulation with M-CSF. At his time point the cells would be normally challenged with RANKL and then differences in RANK expression should have a maximal effect. In fact, ADAM17-deficient cells expressed higher levels of RANK compared to cells from littermate controls (Fig. 4I). Additionally, also CSF1R expression was higher when ADAM17 was lacking (Fig. 4J). These data confirmed that ADAM17 negatively regulates osteoclast differentiation in response to RANKL and M-CSF, most likely by shedding of the receptors for these two mediators.

### ADAM10 inhibition promotes osteoclast differentiation at a later stage

**Fig. 5:**
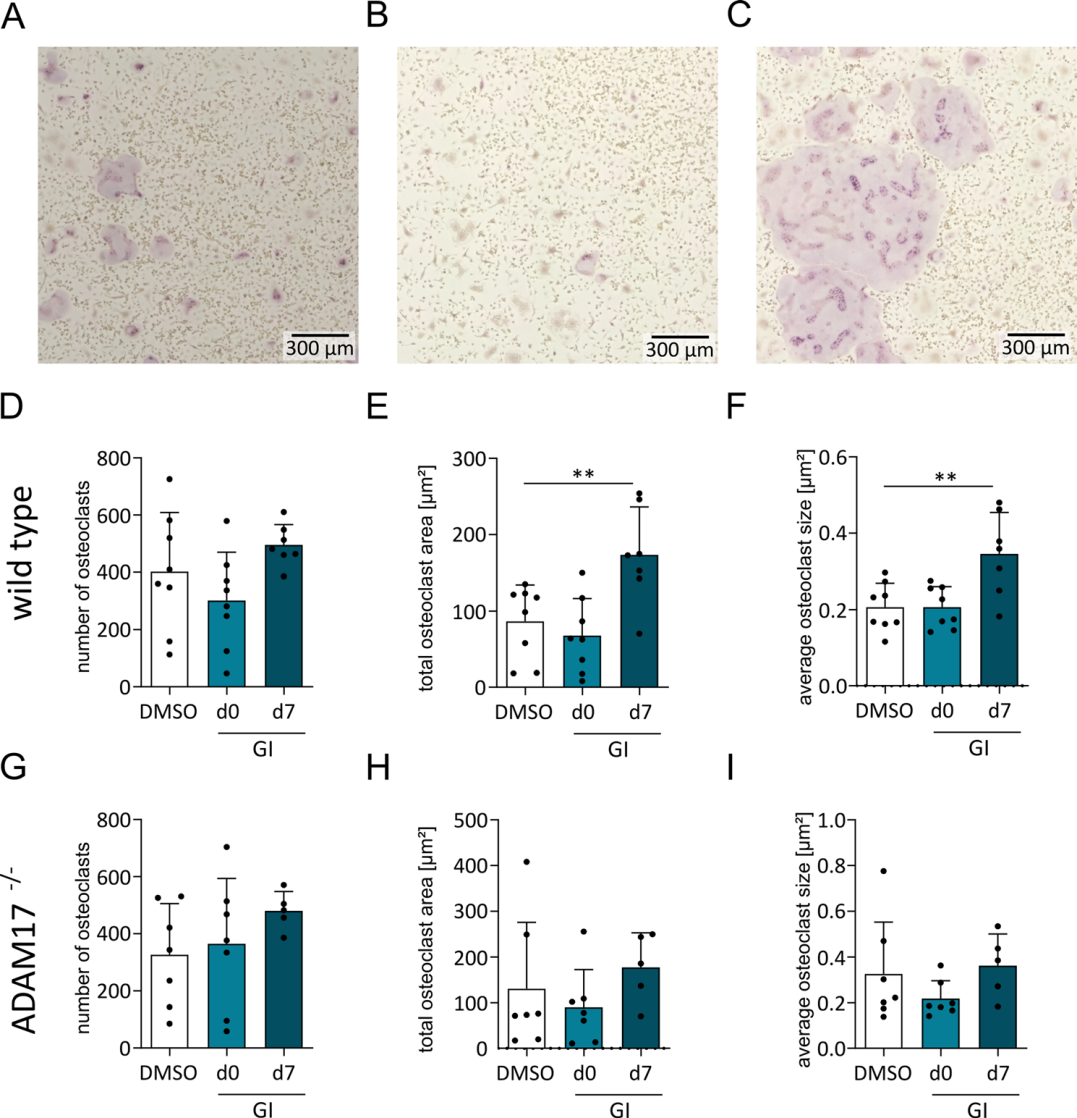
Effect of ADAM10 inhibition on the osteoclastic differentiation of BMDMCs with and without ADAM17 knockout. (**A**–**I**) WT or ADAM17-deficient BMDMCs were treated with GI (10µM) or DMSO together with M-CSF on day 0 or together with RANKL on day 7, and the effect on osteoclast differentiation was analyzed on day 12. Representative images of TRAP-stained WT BMDMCs are shown (**A**–**C**). The number, total area and average size of TRAP-positive cells were determined per image and are displayed as data points for each independent experiment (**D**–**I**). The quantitative data are presented as the means + SD of independent experiments, and significant differences are indicated by asterisks (* *p* < 0.05, ** *p* < 0.01 and *** *p* < 0.001)

To determine the role of ADAM10 it would be ideal to use BMDMCs from mice with specific ADAM10 deficiency in hematopoietic cells. However, culturing BMDMCs from these mice was extremely difficult, and the results were highly variable. For this reason, the contribution of ADAM10 to BMDMC differentiation into osteoclasts was investigated with the specific inhibitor GI. To study time-dependent effects, the inhibitor was added from day 0 when the cells were stimulated with M-CSF or from day 7 when the cells received RANKL. Surprisingly, the osteoclast cell number and size were not changed by inhibition from day 0 on, and there was even a tendency toward reduced osteoclast number and area (Fig. 5A - F). In contrast, the area but not the cell number of osteoclasts clearly increased after inhibition from day 7 (Fig. 5A - F). These findings indicate that during the early stage of stimulation when M-CSF induces proliferation and survival, ADAM10 can increase the number of cells that develop into osteoclasts. In contrast, at a later stage, when RANKL drives terminal differentiation to large multinucleated cells, ADAM10 clearly suppresses the size of osteoclasts.

Finally, to assess a potential additive effect of ADAM17 and ADAM10 we treated ADAM17-deficient BMDMCs with the GI inhibitor. As expected, the average osteoclast size was already higher in the vehicle control with a mean average size of 0.042 mm² compared to the mean average size of 0.026 mm² in the vehicle control of WT BMDMCs (compare Fig. 5F and I). Additional, GI treatment from day 0 on did not change the number or the area of osteoclasts (Fig. 5G-I). Moreover, osteoclastogenesis did not further increase when GI was added beginning on day 7. These results indicate that reducing the activity of either ADAM10 or ADAM17 is sufficient to promote osteoclastogenesis and that this effect is not further enhanced by reduction of both proteinases.

### ADAM10 or ADAM17 inhibition promotes osteoclastic differentiation of human monocytes

**Fig. 6:**
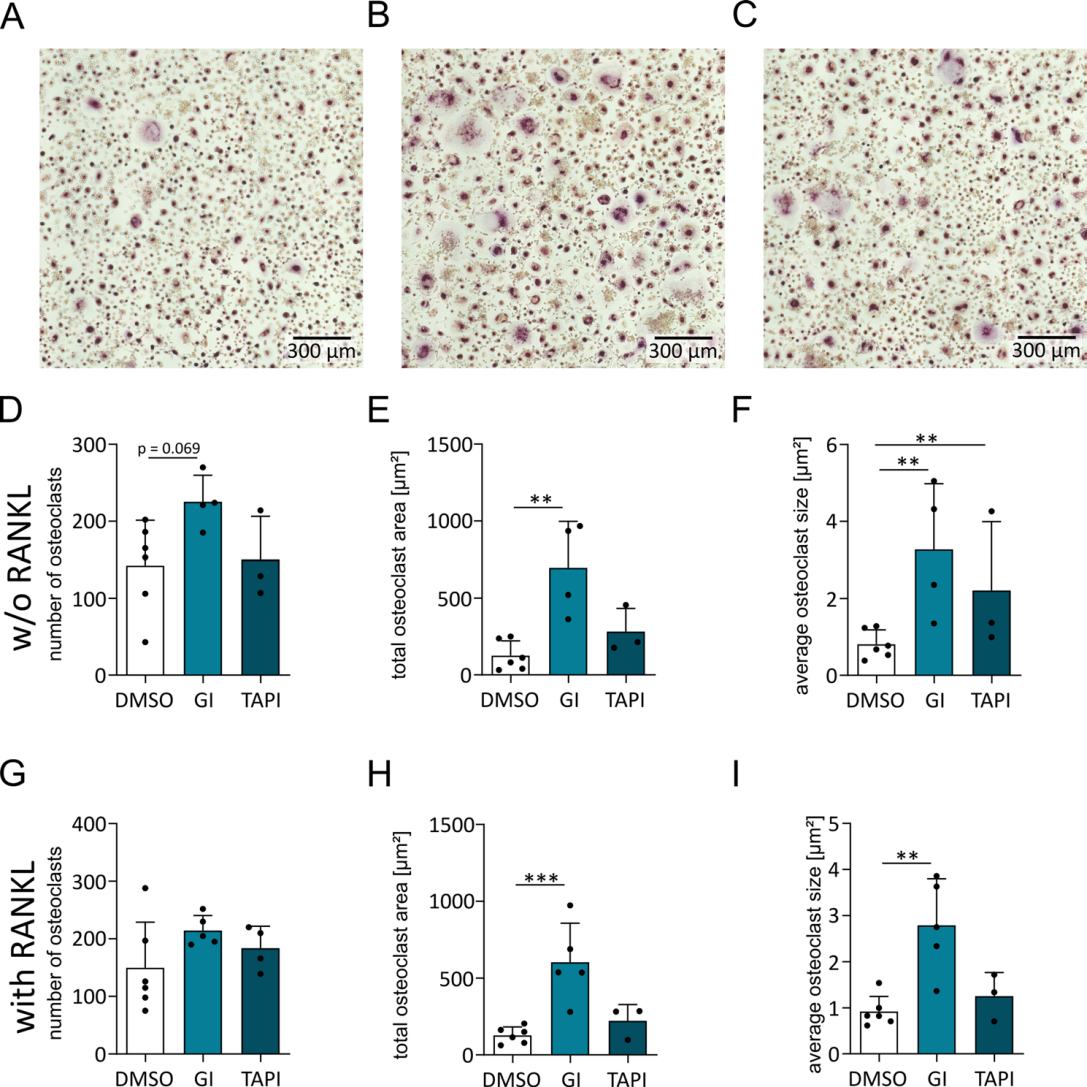
Effect of ADAM10 or ADAM17 inhibition on the osteoclastic differentiation of human PBMCs. (**A**–**I**) PBMCs from healthy donors were treated with 10 µM GI (**A**, **D**, **G**) or 20 µM TAPI (**B**, **E**, **H**) or DMSO (**C**, **F**, **I**) and were stimulated with M-CSF beginning on day 0. On day 7, half of the cells additionally received RANKL (**G**–**I**). On day 17, the effect on osteoclast cell number and area was analyzed. The data are presented as representative images (**A**–**C**) and as the mean + SD (**D**–**I**) of independent experiments. Significant differences are indicated by asterisks (* *p* < 0.05, ** *p* < 0.01 and *** *p* < 0.001)

Since previous experiments were performed with murine cells, we wanted to confirm the relevance of our findings for human cells. We prepared human PBMCs to study the differentiation of human monocytes into osteoclasts in the presence or absence of the ADAM10 or ADAM17 inhibitors GI and TAPI, respectively. PBMCs also contain activated lymphocytes that are known to express RANKL and thereby can promote osteoclast differentiation [35, 36]. We therefore studied whether stimulation with M-CSF alone was sufficient to induce osteoclast differentiation. In fact, we observed osteoclast differentiation already in the absence of exogenous RANKL as indicated by an increase in the number and area of TRAP-positive cells (Fig. 6A – F). This response was clearly enhanced by the ADAM10 inhibitor GI and to some degree also by the preferential ADAM17 inhibitor TAPI. Interestingly, additional stimulation with exogenous RANKL did not further increase osteoclastogenesis. Nevertheless, the effect of the inhibitors on osteoclast size and area remained (Fig. 6G - I). Thus, ADAM10 or ADAM17 inhibition seems to sensitize already unstimulated human mononuclear cell cultures for osteoclast differentiation, which is most likely caused by the presence of lymphocyte-derived RANKL.

## Discussion

The present study provides multiple lines of evidence that the metalloproteinase ADAM17, its regulator iRhom2 and the related proteinase ADAM10 critically influence osteoclast development. This effect was observed in murine RAW264.7 cells, murine BMDMCs and human PBMCs by using either preferential metalloproteinase inhibitors for ADAM10 or ADAM17 or by knockout of ADAM17 or iRhom2. As a possible mechanism, we studied the surface expression of receptors for RANKL and M-CSF which was elevated by ADAM17 knockout or the inhibition of ADAM17 or ADAM10. Thus, our findings clearly indicate a suppressive function of ADAM17, and ADAM10 in osteoclastogenesis which can be conclusively explained by the reduction in sensitivity to M-CSF and RANKL. Osteoclast differentiation was also associated with the downregulation of ADAM10 and ADAM17 as well as the ADAM17 adapter molecule iRhom2 at the mRNA level. Therefore, we propose that this process is part of a feedback loop to enhance osteoclast development.

The role of ADAM17 during bone growth has been studied in previous work, demonstrating that murine chondrocytes require ADAM17 and its regulators iRhom2 and iRhom1 for long bone growth [16–18, 37]. The importance of ADAM17 for the differentiation of the monocytic lineage to osteoclasts has not yet been studied in vivo. It has been reported that ADAM17 deficiency accelerates osteoclastogenesis in vitro [3]. In agreement with this work, we found that ADAM17 inhibition in RAW264.7 cells, or ADAM17 knockout in BMDMCs, increases osteoclast differentiation in vitro. Notably, ADAM17 did not increase the number of osteoclasts but rather the size of multinucleated osteoclasts. This may be due to increased fusion and syncytium formation. Thus, data from pharmacological and genetic manipulation have consistently demonstrated that ADAM17 acts as a negative regulator of osteoclastogenesis in vitro. In line with this finding, knockout of the ADAM17 adapter molecule iRhom2 also increased the formation of osteoclasts in BMDMCs. Interestingly, in contrast to the ADAM17 deficiency, iRhom2 deficiency led to an increase in the number of osteoclasts but not to an increase in the size of osteoclasts. This could be due to the different genetic backgrounds of the mice, as BMDMCs from iRhom2 wild-type mice already produced osteoclasts that were half the size of osteoclasts generated by BMDMCs from ADAM17 wild-type mice. This could also indicate that osteoclastogenesis was simply slower in the iRhom2 mouse strain and that syncytium formation was not yet completed at the time point at which the BMDMCs from the ADAM17 mouse strain already displayed larger osteoclasts. Additionally, the iRhom2-related protein iRhom1 could potentially compensate iRhom2 deficiency and thereby slow down osteoclastogenesis.

It has been reported that endothelial ADAM10 affects osteoclasts at the chondro-osseous junction [19] but the effect of ADAM10 on osteoclast precursors has not been studied before. Pharmacological intervention using GI, which is a well-established preferential ADAM10 inhibitor [29], indicates a role of ADAM10 during RANKL-mediated RAW264.7 cell differentiation into osteoclasts. However, for PBMCs, the situation may be more complex. Surprisingly, we found that ADAM10 inhibition in BMDMCs from wild-type mice decreased numbers as well as the size of developing osteoclasts from an early time point on. When these cells are initially stimulated with M-CSF, the addition of GI limits osteoclast development. Intriguingly, at a later stage, when RANKL was added, GI enhanced osteoclast development. Thus, at an early stage ADAM10 seems to promote the survival of precursor cells, while at a later stage, it counteracts osteoclastogenesis. We cannot rule out that the ADAM10 inhibitor on BMDMC differentiation into osteoclasts was influenced by non-hematopoietic cells in the cell preparation. Additional inhibition of ADAM10 on non-hematopoietic cells may explain the unexpected observation of reduced BMDMC differentiation, when the inhibitor was applied at an early stage. For the studied ADAM17 mediated effects, however, such an indirect influence is unlikely since knockout was cell lineage-specific and thus restricted to hematopoietic cells.

A comparison of the effects of ADAM17 and ADAM10 on osteoclast differentiation revealed that ADAM17 inhibition or ADAM10 inhibition in RAW267.4 cells or PBMCs increased osteoclast differentiation. Interestingly, in RAW267.4 cells ADAM17 inhibition was more effective, whereas in PBMCs ADAM10 inhibition led to a stronger effect. This may suggest that both proteinases can suppress osteoclastogenesis to a different extent dependent on the source of monocytic cells. Moreover, the ADAM17 knockout in BMDMCs led to a relatively similar increase in osteoclast differentiation as the ADAM10 inhibition during a later stage of osteoclast differentiation. Notably, a lack of ADAM10 or ADAM17 activity does not seem to synergize or cause additive effects, as indicated by the combination of ADAM17 knockout and ADAM10 inhibition. This suggests that inactivation of only one proteinase is sufficient to obtain a strong effect on osteoclast differentiation. In other words, both proteinases are required to suppress osteoclast development.

We observed that the downregulation of ADAM17 and ADAM10 mRNA expression was associated with osteoclast development in RAW264.7 cells and BMDMs. Additionally, the mRNA expression of the ADAM17 adapter molecule iRhom2, which is required for the maturation and surface expression of ADAM17, was decreased. These regulatory effects may be due to transcriptional changes or altered mRNA stability. For human peripheral blood monocytes, reduced mRNA expression during osteoclast differentiation has been described for ADAM17 but was not observed for ADAM10 [38]. That we now observed ADAM10 regulation may be due to differences in species or different tissue sources of the monocytic cells. Regardless of whether it is ADAM10, ADAM17, iRhom2 or a combination, a downregulation of these proteins, would reduce the activity of ADAMs, which in turn would promote osteoclast differentiation. It is likely that the downregulation of ADAMs and their adapter molecules is part of a feedback loop for enhancing osteoclast development.

As a potential mechanism involved in the control of osteoclast development, we studied the effects of ADAM17 on RANK and CSF1R shedding. The involvement of ADAM17 and iRhom2 in the shedding of both receptors is well known [3, 4, 39]. Consistent with this knowledge, we found that more receptors are expressed on the cell surface when ADAM17 is inhibited in RAW264.7 cells or when this proteinase is lacking in BMDMCs. A previous study showed that GM-CSF and IL-4 upregulate ADAM17-mediated CSF1R shedding on osteoclast precursors, which is associated with reduced osteoclastogenesis [21]. RANK shedding was also linked to reduced osteoclastogenesis in another study [3]. Thus, it seems very likely that increased densities of both receptors RANK and CSF1R lead to an increased sensitivity to their ligands and therefore increased osteoclast development by RANKL and M-CSF when ADAM17 activity is lacking.

CSF1R shedding from the cell surface can be suppressed not only by ADAM17 knockout or inhibition but also by ADAM10 inhibition. Moreover, ADAM10 inhibition tends to reduce RANK shedding. Hence, both receptors could represent new relevant substrates for ADAM10. The proteinase is known to shed RANKL and is thereby involved in osteoclast differentiation [20] but ADAM10 has not yet been described as a shedding proteinase for RANK on osteoclasts themselves. Notably, several other surface molecules such as CX3CL1, IL6R, CD44, Notch and ACE2 can be shed by ADAM10 in addition to ADAM17. Interestingly, constitutive shedding is in many cases mediated by ADAM10 while shedding by ADAM17 is more predominant after cell stimulation [9, 40, 41]. Often, ADAM17-mediated shedding can be provoked by the activation of PKC with PMA. In the case of RANK and CSF1R on macrophages, both ADAM10 and ADAM17 seem to contribute to shedding, and no further cell activation is necessary. However, it is still possible that cleavage can be further enhanced by cell stimulation. Additional studies are required to characterize the cleavage of both receptors by ADAM10 and ADAM17 in more detail, including studies on stimulatory conditions, kinetics, and cleavage sites.

Since ADAM10 inhibition can reduce the shedding of CSF1R and, to some degree, RANK, it is possible that ADAM10 can interfere with osteoclast differentiation in a similar way as ADAM17. This may be the case when the ADAM10 inhibitor is added at a later phase of osteoclast differentiation when cells are stimulated with RANKL. It could be that the inhibition of RANK shedding during this phase enhances the response to RANKL, which could explain the enhanced osteoclast differentiation. Surprisingly, we observed that ADAM10 inhibition at an early stage when M-CSF was added led to slight suppression of osteoclast development. This finding argues against the relevance of suppressed CSF1R shedding in this situation, which would increase the responsiveness for CSF1R. Importantly, ADAM10 can also shed other surface molecules that are implicated in osteoclast development. For example, Notch is shed and activated via ADAM10. Downregulation of Notch activation inhibits osteoclastogenesis in vitro [42]. The IL6 receptor on macrophages is shed by ADAM17 and trans-signaling via the soluble receptor can promote osteoclastogenesis [43]. Moreover, EGFR ligands are shed by ADAM10 and ADAM17 and can influence osteoclast differentiation potential in many ways or promote the survival of differentiating osteoclasts [44, 45]. It may also be possible that ADAM10 and ADAM17 shed cell adhesion molecules that are implicated in contact formation during the fusion of osteoclasts.

The observed positive effect of ADAM10 or ADAM17 inhibition or knockout on osteoclast differentiation raises the question of whether these interventions may lead to increased bone resorption in vivo. To date, such effects have not been described in the literature. Of course, ADAM proteinases on different cell types other than osteoclasts would influence osteoclastogenesis in vivo, for instance, through ADAM-dependent shedding of RANKL on osteocytes or osteoblasts, chondrocyte-bone crosstalk or bone vascularization [16–20]. Thus, a detailed investigation of conditional knockout mice lacking the proteinases, specifically in monocytic osteoclast precursor cells, is needed. In humans, very seldom mutations in the ADAM17 gene can cause functional ADAM17 deficiency. These patients have recurrent infections due to the loss of tumor necrosis factor α (TNFα) shedding, but the effects on hard tissues have not yet been reported [46]. It could well be that the influence of ADAMs on bone resorption becomes visible only under pathological conditions of bone remodeling.

ADAM10/17 inhibition is considered as therapeutic strategy to limit inflammatory responses or tumor growth induced by the shedding of TNFα or growth factors, respectively. For broad-spectrum inhibitors of metalloproteinases, musculoskeletal or hepatotoxic side effects have been noted in early clinical trials [47]. Additionally, the more specific ADAM inhibitors should be carefully checked for side effects including any undesired increase in bone resorption. Vice versa, the activation of ADAMs could limit bone resorption. This may be achieved by specific stimuli that promote enhanced ADAM expression and activity [21, 48] or by promoting the upregulation of ADAM surface expression via the induction of adapter molecules such as iRhoms [11].

### Electronic supplementary material

Below is the link to the electronic supplementary material.

Supplementary Material 1

## Data Availability

No datasets were generated or analysed during the current study.
