# Supplementary material for: Downregulation of the metalloproteinases ADAM10 or ADAM17 promotes osteoclast differentiation
Source: Cell Commun Signal. 2024 Jun 11;22:322. doi: 10.1186/s12964-024-01690-y (PMC11167776; doi:10.1186/s12964-024-01690-y)
Supplement: Supplementary file 1 — Supplementary Material 1 [file 12964_2024_1690_MOESM1_ESM.docx]

**Suppl. Table 1: Sequences and conditions for the qPCR primers used.**

| Gene | Oligonukleotide  sequence | Annealing temperature |
| --- | --- | --- |
| *mAcp5* | *Forward:* CCT GAG ATT TGT GGC TGT G  *Reverse:* GTC TCC TGG AAC CTC TTG TC | 61 °C |
| *mAdam10* | *Forward:* AGC AAC ATC TGG GGA CAA AC  *Reverse:* TGG CCA GAT TCA ACA AAA CA | 57 °C |
| *mAdam17* | *Forward:* AAA CCA GAA CAG ACC CAA CG  *Reverse:* GTA CGT CGA TGC AGA GCA AA | 57 °C |
| *mCtsk* | *Forward:* AGA AGC AGT ATA ACA GCA AGG  *Reverse:* TTC TTC ACT GGT CAT GTC TC | 61 °C |
| *mEef2* | *Forward:* TC ACAA TCA AAT CCA CCG CC  *Reverse:* ATG GCC TGG AGA GTC GAT GA | 60 °C |
| *mGapdh* | *Forward:* CAT GGC CTT CCG TGT TCC TA  *Reverse:* ACT TGG CAG GTT TCT CCA GG | 60 °C |
| *mMmp9* | *Forward:* GTA TCT GTA TGG TCG TGG CT  *Reverse:* CGT GGG AGG TAT AGT GGG A | 61 °C |
| *mNfatc1* | *Forward:* TAT ATG AGC CCA TCC TTG CC  *Reverse:* AGC CGT CCC AAT GAA CAG | 61 °C |
| *mRhbdf2* | *Forward: AGA GCG TGA AGT ACA TCC*  *Reverse: TAA AGT CTC CGA GCA GTC C* | 60 °C |
| *mRps29* | *Forward:* CCT TTC TCC TCG TTG GGC G  *Reverse:* GAG CAG ACG CGG CAA GAG | 61 °C |


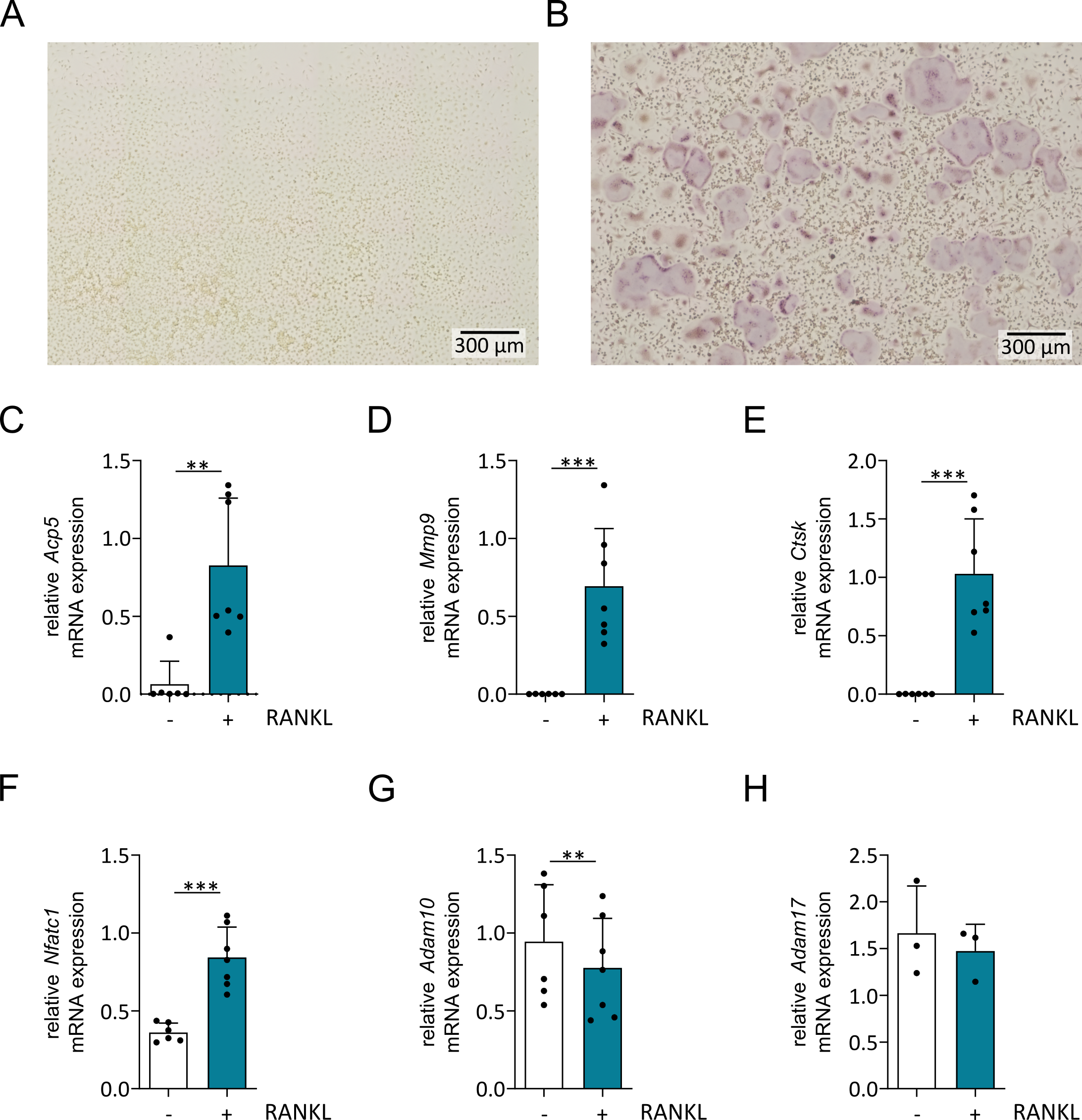


**Suppl. Figure 1: Osteoclastic differentiation of BMDMCs from wild-type Vav-Cre ADAM17 mice.**

A–B) Isolated BMDMCs from WT Vav-Cre ADAM17 mice were differentiated into osteoclasts by stimulation for 7 d with M-CSF and subsequently for 5 d with additional RANKL. Cells that were only treated with M-CSF served as a control. After 12 days, the cells were stained for TRAP. Representative images of TRAP-stained murine BMDMCs treated with M-CSF (A) or with both M-CSF and RANKL (B) are shown. D-I) The mRNA expression of the osteoclast-associated marker genes Acp5/TRAP (D), Mmp9 (E), Ctsk (F) and Nfatc1 (G) as well as that of Adam10 (H) and Adam17 (I) was determined by qPCR. The expression of these genes was related to that of the reference genes Gapdh and Rps29. The quantitative data are presented as the means +SD of independent experiments, and significant differences are indicated by asterisks (* p < 0.05, ** p < 0.01 and *** p < 0.001).


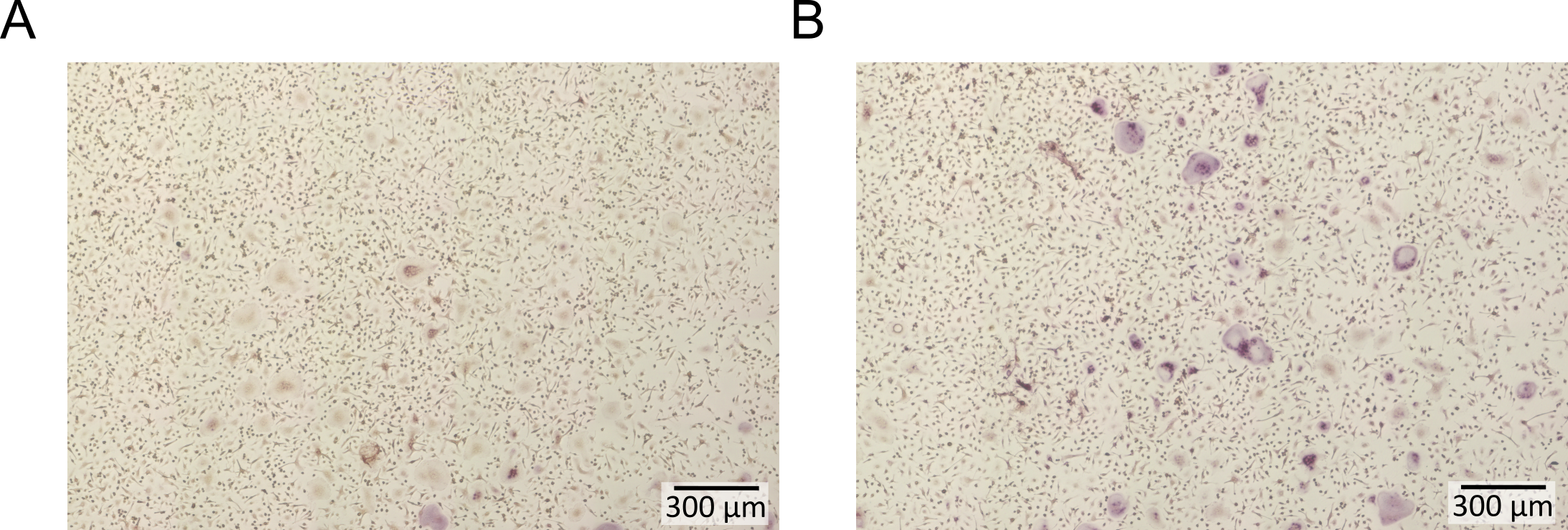


**Suppl. Figure 2: Osteoclastic differentiation of BMDMCs from iRhom2 knockout mice.**

A, B) WT or iRhom2-deficient BMDMCs were stimulated with M-CSF on day 0 and subsequently with additional RANKL on day 7. After day 11, the cells were fixed and stained for TRAP. Representative images of the osteoclastic differentiation of WT (A) and iRhom2-deficient BMDMCs (B) are shown.
